# Development and Validation of Radiographic Measurement Templates for Standardized X-ray Analysis

**DOI:** 10.7759/cureus.83232

**Published:** 2025-04-30

**Authors:** Alqasim Elnaggar, David Allen, David Abdelnour, Magd Boutany, Ahmad Almaat, Andrew Knapp, Cameron J Hoard, Rahul Vaidya

**Affiliations:** 1 Department of Orthopaedic Surgery, Wayne State University School of Medicine, Detroit, USA; 2 Department of Orthopaedic Surgery, Wayne State University Detroit Medical Center, Detroit, USA

**Keywords:** bonesetter app, digital sizing, digital templating, magnification correction, pre-op planning, x-ray analysis

## Abstract

Introduction: Accurate radiographic measurements are crucial for musculoskeletal diagnosis and surgical planning. However, the absence of standardized scaling markers in many radiographs can lead to measurement errors and variability. Template-based scaling offers a reliable solution when traditional markers are unavailable. This study describes the development of 21 anatomical templates designed to enable accurate radiographic scaling by accounting for magnification effects, providing a standardized measurement method for clinical and research applications.

Methods: 1,050 radiographs from a level 1 trauma center were analyzed to develop templates for 12 anatomical regions across various imaging views. Each template was generated by averaging measurements from 50 images per anatomical site. Magnification correction factors were calculated by determining the measurement discrepancy in radiographs containing objects of known size. The templates were then integrated into the DetroitBonesetter (DBS) software and validated by comparing scaled measurements with reference objects such as implanted plates and screws.

Results: A total of 21 radiographic templates were developed. Each template was corrected for magnification using implanted objects of known size. Validation testing was performed by comparing scaled measurements against these known references, with all templates achieving over 90% accuracy. The highest mean accuracy was 97.42%, and the lowest was 92.86%. The validated templates included the wrist (anteroposterior (AP)/lateral (Lat)), ankle (AP/mortise/Lat), knee (AP/Lat), proximal tibia (AP), tibia (AP/Lat), proximal humerus (AP), humerus (AP/Lat), distal humerus (AP/Lat), shoulder (AP/Lat), distal femur (AP/Lat), elbow (Lat), and olecranon (Lat). The templates were integrated into the DBS software for use in clinical and research settings.

Conclusion: The template-based scaling method provides a standardized and magnification-corrected approach to radiographic measurements. These templates enhance a user's ability to make measurements in the absence of scaling markers when involved in orthopedic surgical planning, research, and education.

## Introduction

Radiographic assessment is an indispensable tool in musculoskeletal diagnosis and treatment planning. Accurate measurements are essential for evaluating fractures, assessing alignment, and planning surgical interventions. Standardized measurement tools, such as sizing coins or rulers, are often included in radiographs to enable accurate scaling. However, in clinical practice and research settings, radiographs frequently lack these embedded markers, thereby limiting measurement precision and introducing variability [1]. 

Measurement errors in radiographic analysis can have significant clinical implications. Overestimation or underestimation of fracture displacement, implant sizing errors, or inaccurate joint space assessments can lead to inappropriate treatment decisions. Consequently, when direct scaling tools are unavailable, alternative methods are essential for ensuring relatively reliable measurements. When used properly, free online software such as DetroitBonesetter (DBS), which is a third-party picture-archiving and communication system (PACS), allows for radiographic analysis and measurements to be made in the pre-operative planning steps [2,3]. DBS has demonstrated accuracy in its application to the field of arthroplasty and revealed no significant difference between templated and implanted femoral components [4]. It has also been shown to be an effective tool in the education of orthopaedic surgeons, allowing them to learn skills such as axial alignment and address miscalculations [5]. Template-based scaling has emerged as a valuable alternative when traditional markers are unavailable. Studies have demonstrated the effectiveness of morphometric analysis in generating standardized anatomical templates derived from population-based data, providing dependable results when correction factors are appropriately applied [1,6].

In orthopaedic surgery, digital templating has become a widely used method for preoperative planning, allowing surgeons to determine optimal implant size and alignment, thus reducing operative times and potential complications [7]. The accuracy and reliability of digital templating have been extensively studied, showing high intra- and inter-observer consistency, particularly for predicting prosthesis sizes in hip and knee arthroplasty. For instance, digital templating has demonstrated accuracy rates as high as 89% for cemented femoral stems within one prosthesis size [8]. Expanding these principles toward creating templates for general radiographic measurement may offer similar advantages in clinical and research settings while maintaining safety [6].

Radiographic magnification, however, poses a significant challenge for accurate scaling as it varies between imaging protocols and anatomical regions. Varying degrees of magnification are influenced by patient positioning, beam angulation, and source-to-image distance [9]. Given the limitations associated with physical markers, alternative calibration methods have been evaluated. Previous research has suggested the use of a low-cost 1-inch ball bearing, taped to the inner thigh at the height of the femoral head, as a method to scale images and account for magnification during the preoperative planning of hip arthroplasties, which require accurate implant measurement [10]. Others suggest that employing a standardized magnification factor when planning for hip hemiarthroplasty, such as a fixed 120%, is often more reliable than markers, providing accurate implant predictions even without direct calibration tools [7,11].

This report describes the development of standardized templates based on averaged dimensions from 50 radiographs per anatomical site and aims to provide an effective, scientifically validated alternative for accurate measurement where traditional sizing markers are unavailable. A total of 1,050 radiographic images were analyzed to create 21 templates, ensuring robust data collection and enhanced reliability. The process of calculating, outlining, and correcting for variations in magnification is described and offers suggestions for the practical use and application of these templates in scaling images during clinical, educational, and research settings.

## Materials and methods

**Figure 1:**
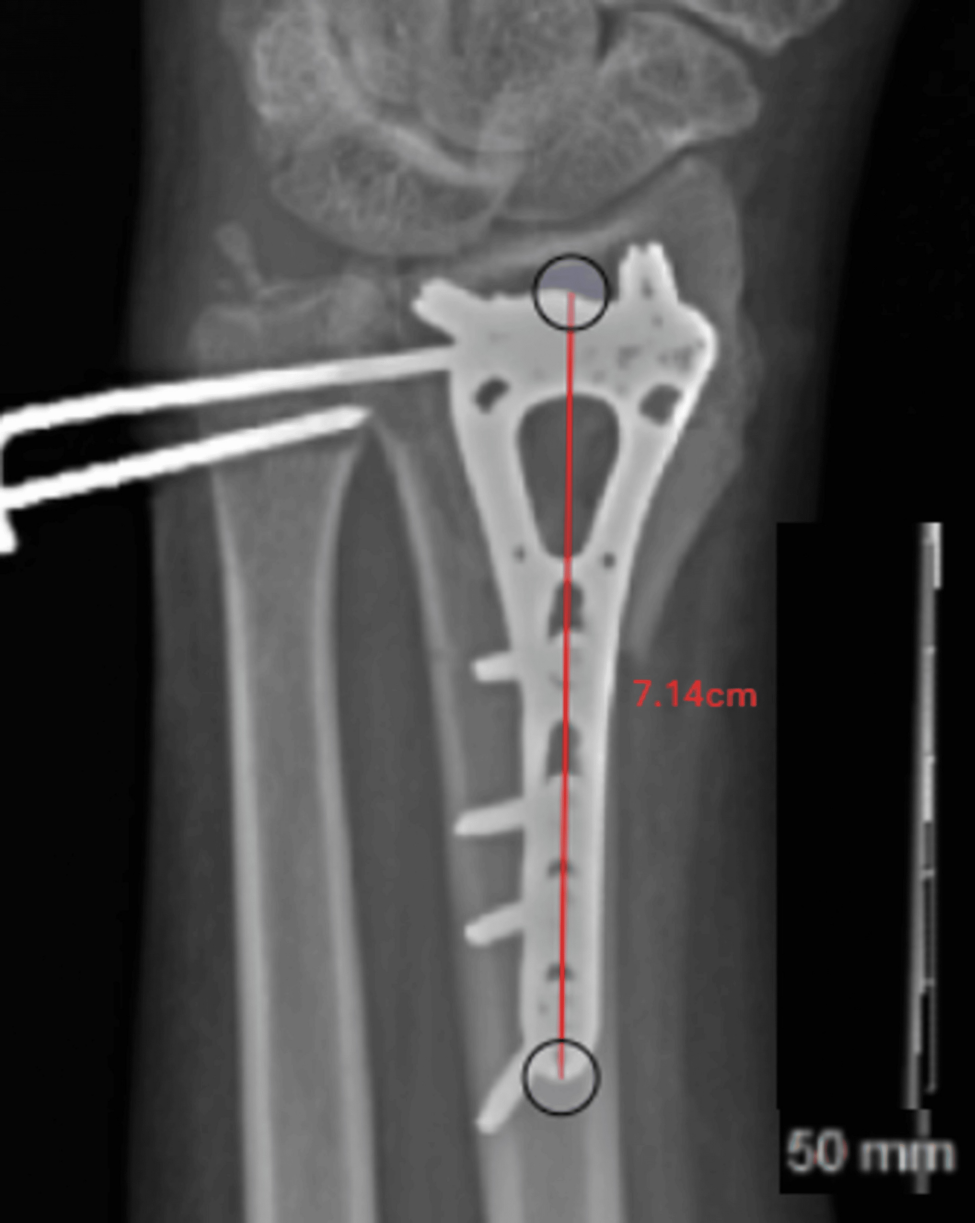
Calculation of the magnification factor A 68-mm volar wrist plate scaled using the 50-mm ruler included in the radiograph is measured to be 71.4 mm, revealing a magnification of 105%. Measured in the DetroitBonesetter (DBS) software.

We conducted an IRB-approved analysis of 1,050 radiographic images from a level 1 trauma center, ensuring that 50 images represented each anatomical site. Twenty-one templates were developed for 12 anatomical regions and views, such as the anteroposterior (AP), mortise, and lateral (Lat) views. Each template was generated by averaging anatomical dimensions across 50 images per anatomical site and view. Images were sourced from healthy adult (18-60-year-old) male and female patients with no injury to the measurement site, and patient positioning was consistent for each region. Height and BMI were not considered to allow the templates to be more generalizable across these demographics. Correction factors were calculated and applied to the averaged measurement to address the magnification effects inherent in radiographic imaging. Plates or nails of known sizes, which are at the same level as the anatomical site of interest and magnified to the same degree, were measured after being scaled to a ruler included in the radiograph. This allowed for a determination of the degree of magnification. For example, when using a standard radiographic ruler to scale the image, a volar wrist plate, which was confirmed to be 68 mm through the review of a patient’s operative note, was measured to be 71.4 mm on DBS, as shown in Figure 1. This suggests that the AP view of the wrist demonstrates a magnification factor of 105%, requiring a 5% reduction in template dimensions to ensure accuracy. This was repeated for each site, and the following correction factors were applied to adjust the average size of each template. The wrist, ankle, and tibia demonstrated 105% magnification, necessitating a reduction of 5%. The elbow, olecranon, humerus, and shoulder exhibited 107% magnification, necessitating a reduction of 7%. Finally, the femur and knee revealed 108% magnification, necessitating a reduction of 8%.

**Figure 2:**
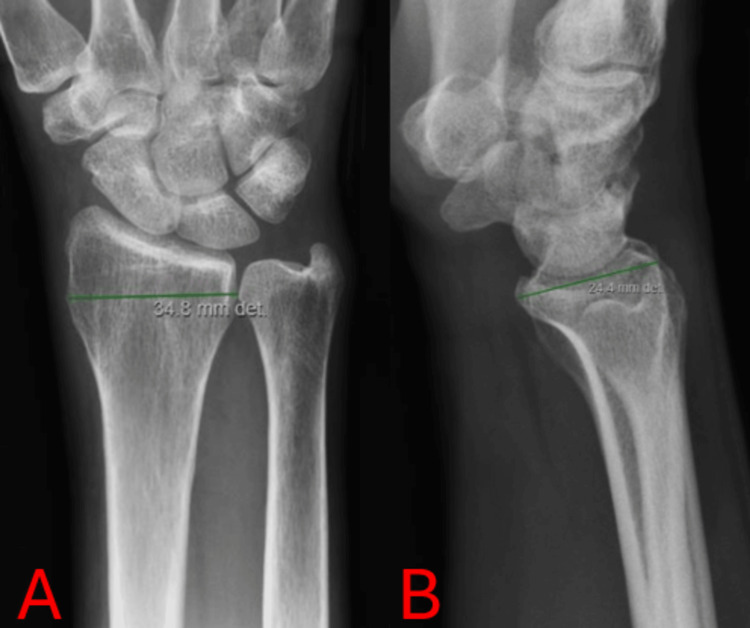
Initial measurements Panel A illustrates the transverse diameter of the distal radius measured in the AP view. Panel B demonstrates the anteroposterior diameter measured in the lateral view. Similar measurements were repeated on 50 different radiographs of the wrist, as well as each anatomical site, to produce 21 average values. Measured on the picture-archiving and communication system (PACS) in patient records.

To generate the templates, measurements were first taken from each anatomical site by identifying key anatomical landmarks, as demonstrated in Figure 2. For example, in the wrist AP template, the transverse diameter, which represents the largest width of the distal radius transversely, was selected as the site of measurement [12]. These values were then averaged across the 50 radiographs for each anatomical region. The distal radius AP template demonstrated an average measurement of 36.33 mm and was adjusted to 34.5 mm after accounting for the 105% magnification factor. Similarly, the lateral measurement of the distal radius was adjusted from 25.28 mm to 24.02 mm after correction.

## Results

**Figure 3:**
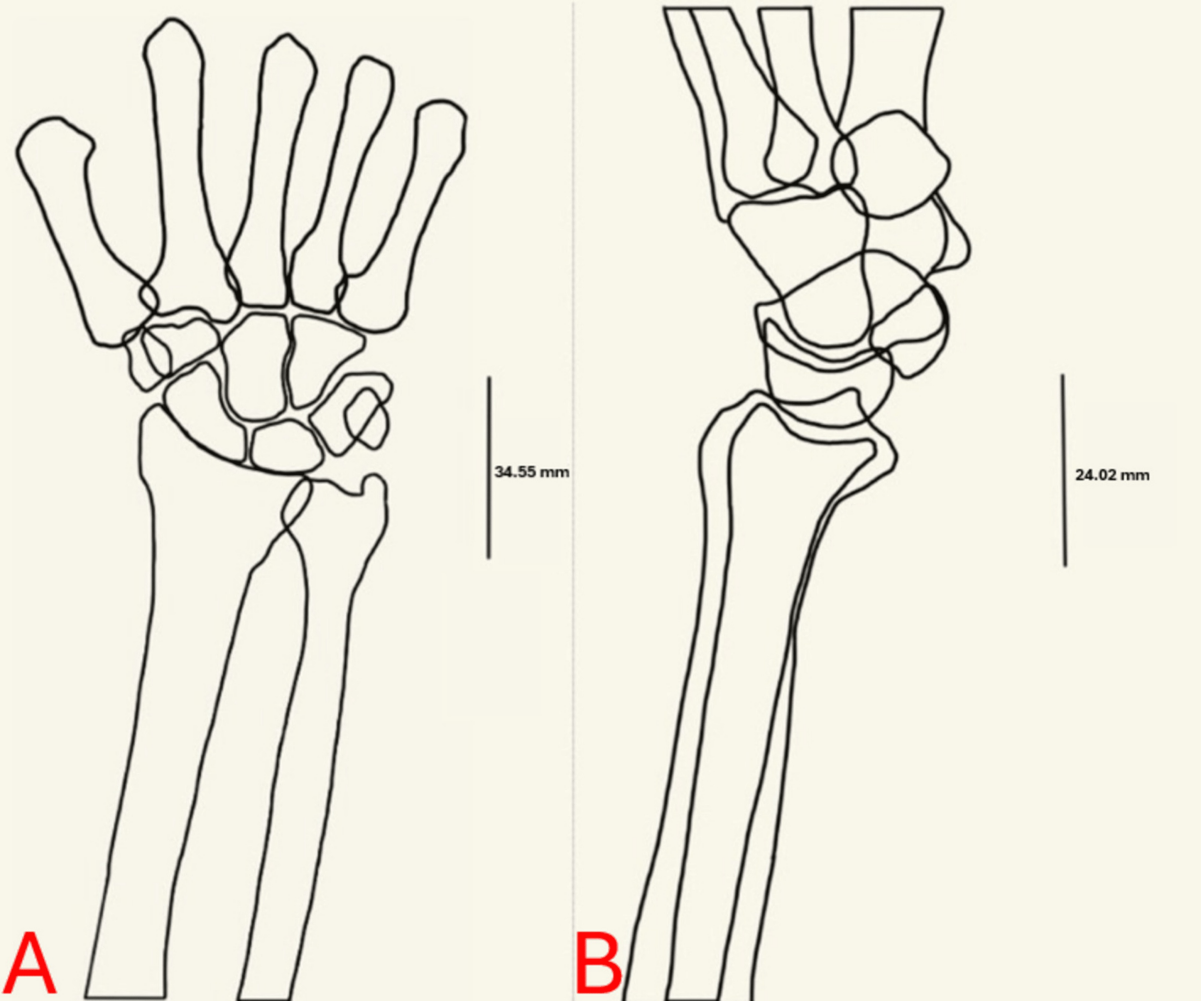
Programmed templates Panel A visualizes the AP wrist template. Panel B visualizes the lateral wrist template. These were developed by tracing a representative scan. Lines were included with measurements that corresponded to the corrected averages to allow for simplicity in scaling the images when programming them into the DBS program. Twenty-one templates in total were created. Figure credits: Alqasim Elnaggar. Templates were made by tracing the radiographs on a tablet.

The measurement process was repeated, yielding an average measurement derived from 50 radiographs for each anatomical site and view. Correction factors were applied appropriately to ensure that magnification was taken into account for each template. A representative radiograph was selected for each site, traced by hand, and scaled to match the averaged and corrected dimensions. This method formed the final templates, such as those shown in Figure 3, which were programmed for use in DBS. The templates that were uploaded for use include the wrist (AP/Lat), ankle (AP/mortise/Lat), knee (AP/Lat), proximal tibia (AP), tibia (AP/Lat), proximal humerus (AP), humerus (AP/Lat), distal humerus (AP/Lat), shoulder (AP/Lat), distal femur (AP/Lat), elbow (Lat), and olecranon (Lat) templates. They are accessed through the function that allows for scaling a radiograph relative to another object in DBS.

**Figure 4:**
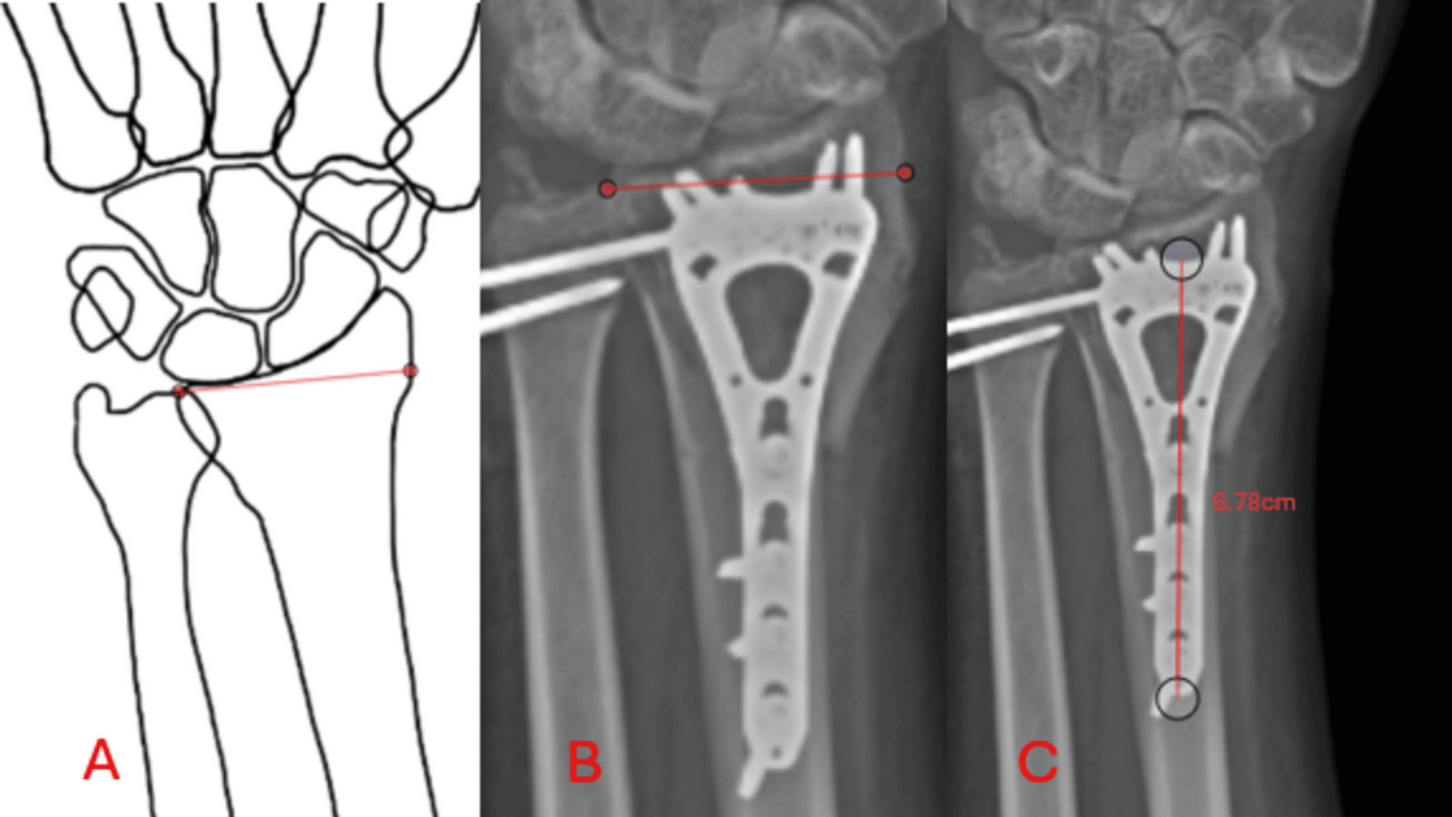
Template validation Panel A demonstrates the selection of two points on the template. Panel B demonstrates the selection of two points on the radiograph, corresponding to the same locations on the template. Panel C demonstrates the automatic calculation of the length of a line made on the radiograph. This is demonstrated by the measurement of a 68-mm volar wrist template, to ensure that the measurement is validated at a comparable level of magnification as the wrist, yielding a 99.7% accurate result (6.78 cm/6.8 cm = 0.997). Figure credits (Panel A): Alqasim Elnaggar. Templates were made by tracing the radiographs on a tablet.

Templates were subsequently programmed into the DBS software and are accessible to users when they upload a radiograph for analysis. When they select the option to scale their radiograph relative to another object, the templates are available in a drop-down menu, allowing the user to select one that corresponds to the relevant anatomical site. To validate the templates, radiographs containing fracture plates or nails of known sizes were uploaded into DBS. When validating the wrist template, a volar wrist plate was used as a point of comparison to ensure that the measurements were made at similar levels of magnification, rather than a sizing coin, which is typically at a lesser distance from the x-ray receiver and will not accurately account for magnification [7]. By selecting corresponding points on the template and the radiograph of interest, the software automatically scaled the radiograph accordingly. The line function within DBS, which can now display measurements scaled to the template, was used to calculate the length of the plate, as shown in Figure 4. The measured length was subsequently compared to the true length, and the accuracy was recorded. This was repeated five times for each template, and the accuracy was averaged to provide a mean for each template. All templates achieved higher than 90% mean accuracy, with the greatest being 97.42%. Table 1 demonstrates the average accuracy achieved for each template.

**Table 1:** Template validation Table demonstrates the results of validating each template against objects of known sizes. Accuracy is presented as a percentage. AP: anteroposterior view; Lat: lateral view; Mortise: mortise view; Std. Deviation: standard deviation; Std. Error: standard error

Template Validation				
Template	Sample Size	Accuracy Mean	Accuracy Std. Deviation	Accuracy Std. Error
Ankle AP	5	97.42%	0.01701	0.00761
Wrist AP	5	96.90%	0.02066	0.00924
Distal Humerus Lat	5	96.38%	0.02663	0.01191
Ankle Lat	5	96.28%	0.01489	0.0666
Wrist Lat	5	96.20%	0.01181	0.00528
Shoulder Lat	5	96.06%	0.02077	0.00929
Ankle Mortise	5	95.96%	0.01905	0.00852
TIbia AP	5	95.56%	0.02663	0.01191
Distal Femur AP	5	95.42%	0.0166	0.00743
Tibia Lat	5	95.34%	0.01674	0.00749
Proximal Tibia AP	5	95.28%	0.01524	0.00681
Shoulder AP	5	95.28%	0.041707	0.01837
Humerus Lat	5	95.20%	0.03073	0.01374
Knee Lat	5	95.18%	0.02292	0.01025
Distal Femur Lat	5	95.14%	0.01868	0.00835
Knee AP	5	94.92%	0.02799	0.01252
Proximal Humerus AP	5	94.90%	0.02456	0.01098
Distal Humerus AP	5	94.86%	0.0251	0.01122
Olecranon Lat	5	94.48%	0.02577	0.01153
Elbow Lat	5	93.60%	0.02456	0.01098
Humerus AP	5	92.86%	0.06334	0.02833
Total	105	95.39%	0.02435	0.01373

## Discussion

Overall, accurate radiographic measurements are essential in clinical and research applications. Sizing markers, such as coins or radiographic rulers, are not always used when radiographs are captured or may be omitted from certain images, such as those used in research or education. This affects the physician or observer’s ability to measure, prepare, and study different pathologies. The lack of sizing marker availability in an image invites many other techniques to be employed [10,11]. For instance, there is fixed magnification scaling, which has been explored and applied to hip hemiarthroplasties and has demonstrated a safe degree of measurement in certain settings, such as femoral head measurement [10]. Other studies suggest that fixed magnification methods were more reliable and efficient than using calibration balls in the planning of total hip replacement components [1]. We show in this report that template-based scaling is adaptable and takes into account anatomical variation. The template-based scaling method is useful because it creates a standard that has been corrected for varying degrees of magnification across several anatomical sites and views and is applicable when the sizing markers are improperly placed or unavailable. Ensuring that magnification is accounted for is vital, as evidence has pointed toward higher rates of intraoperative fractures when a radiograph was magnified more than the template [13].

Template-based scaling is an effective methodology for scaling radiographs. Our templates demonstrated up to 97.42% accuracy when validated against fracture plates of known sizes that were included within the image. The template at the lower bound, humerus AP, held an accuracy of 92.86%, which is higher than some accuracies that have been deemed acceptable in prior studies, such as that by Smith et al., which revealed 89% of the cemented femoral stems being within one prosthesis size when using digital templating [8]. This method of validation eliminates the possibility of a discrepancy due to magnification, as opposed to when compared to a sizing coin or radiographic ruler, where the distance of the reference marker to the X-ray detector is much less than the anatomical site of interest. A coin would be significantly closer to the X-ray detector than the surface of the distal radius and will not be magnified to the same degree as the surface of the radius or other anatomical sites. Other studies have tried to account for magnification by placing a sizing coin on a platform and raising it to the proper anatomical level [14]. In our process, the need to measure a sizing coin elevated to the level of the bone was eliminated, by using images with plates or screws placed in the anatomical site and comparing the measured size to the known size to determine the magnification factor. Scaling a radiograph to an object of known size is the purpose of a sizing coin, so fracture plates effectively replace the need for them. Their sizes are always recorded, and they are already at the proper level to ensure equal magnification between them and the anatomical site. However, they are not available as a method of scaling preoperatively and can only be used to calculate the magnification factor and validate the template's measurements.

Limitations to the template-based scaling we presented include the fact that it does not account for variability in anatomic dimensions due to variations in local populations. Therefore, our template may work for the Detroit area, but it may not be generalizable to other regions. Evidence has pointed towards variations in bone dimensions across ethnicities and genders, which may impact the creation of a template by averaging dimensions [15]. Additionally, there is a lack of templates for specific anatomical sites, but in our study, we created 21 different templates in hopes of rectifying this limitation. Template-based scaling is meant to ensure consistency and reduce variability, giving physicians and researchers a measurement that can be worked with in scenarios where sizing markers are unavailable. This manuscript provides guidance and outlines the methods used for the creation of anatomical templates in hopes of expanding the use and availability of a template-based scaling system across anatomical sites, radiographic views, age groups, genders, and more.

## Conclusions

The development of 21 standardized templates offers a practical solution for achieving relatively reliable measurements in the absence of sizing markers. By incorporating averaged dimensions and correcting for magnification errors, these templates provide working accuracy and reproducibility in musculoskeletal radiographic assessments. The inclusion of 1,050 radiographic images ensures robust data collection, enhancing template reliability.

These templates have broad applications in orthopaedic research, resident education, and surgical planning. The DBS software’s ability to provide accurate scaling in the absence of sizing markers, through the implementation of these templates, presents a valuable tool for clinical and research environments alike. Future reproductions should expand template development across broader populations and anatomical regions. Additionally, further refinements in image processing techniques and software automation could enhance the utility of these templates, making them more accessible for widespread use in orthopaedic and radiographic studies.
